# Recent Progress in Bismuth Vanadate-Based Photocatalysts for Photodegradation Applications

**DOI:** 10.3390/nano15050331

**Published:** 2025-02-21

**Authors:** Yangyang Zhang, Hao Li, Dan Yin

**Affiliations:** School of Ecology and Environment, Zhengzhou University, Zhengzhou 450001, China; zyy2487996926@163.com (Y.Z.); lihao2010@zzu.edu.com (H.L.)

**Keywords:** BiVO_4_, photocatalysis, organic pollutants, degradation

## Abstract

Bismuth vanadate (BiVO_4_), a well-known semiconductor photocatalyst with various advantages, has shown great potential in addressing energy and environmental issues. However, its inherent drawbacks restrict the photocatalytic performance of pure BiVO_4_. In the past few years, many efforts have been devoted to improving the catalytic activity of BiVO_4_ and revealing the degradation mechanism in depth. In this review, we summarized the recent progress on BiVO_4_ in the field of photocatalytic degradation, including the strategies which enhance light absorption ability and suppress the recombination of charge carriers of BiVO_4_, as well as the related degradation mechanism. Finally, future prospects and challenges are summarized, which may provide new guidelines for designing more effective BiVO_4_-based photocatalysts for the degradation of persistent organic pollutants.

## 1. Introduction

With the ever-growing energy crisis and environmental pollution, there is a pressing need for developing effective methods to solve the above issues [1,2,3]. Among various techniques, photocatalysis is considered as one green and eco-friendly method that could utilize solar energy for the removal of harmful pollutants [4,5,6]. Photocatalysts, as the core of photocatalytic reactions, have drawn much attention for practical applications.

**Figure 1:**
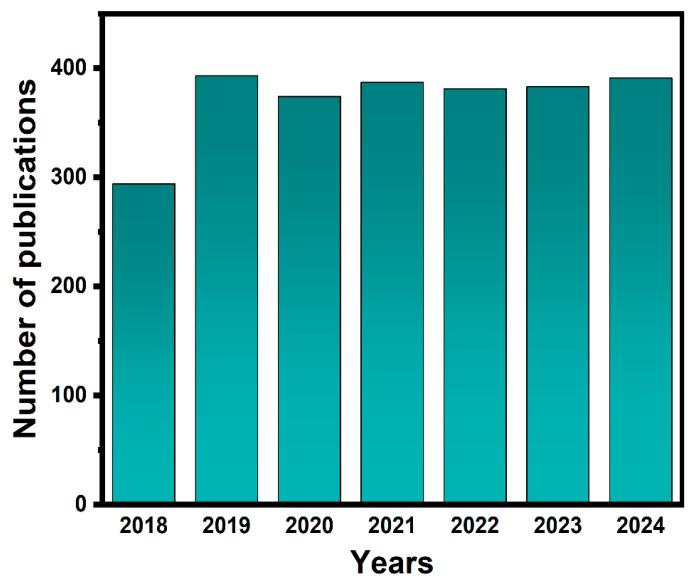
Number of publications for BiVO_4_-based photocatalyst material in recent years (2018–2024). Key words: BiVO_4_ photocatalytic; source: Web of Science.

Among all photocatalysts, titanium dioxide (TiO_2_) stands out as one of the widely utilized semiconductors for photocatalytic applications. However, pure TiO_2_ exhibits photoactivity only under ultraviolet (UV) radiation, which accounts for only approximately 5% of natural sunlight [7]. The photocatalytic performance of TiO_2_ is limited by a large band gap and the high rate of charge recombination. BiVO_4_ has emerged as one promising famous n-type semiconductor for photo(electro)catalytic applications, especially the photocatalytic degradation of organic pollutants, due to its unique electronic structure and outstanding optical properties [8,9,10]. The electronic structure of BiVO_4_ is composed of Bi 6s and O 2s orbitals. The extra Bi 6s orbital will shorten the transfer distance of the excited electrons in the valance band of BiVO_4_ to the V 3d state available in the conduction band of VO_4_^3−^, which may cause a small band gap in BiVO_4_ and extend the light absorption region [11]. The flexible optical characteristic, with a band gap of ~2.4 eV, and the good chemical photostability of BiVO_4_ make it popular in the field of photocatalytic degradation [12,13,14]. Since Kudo et al. investigated the photocatalytic activity of BiVO_4_ in the year 1999 [15], much more attention has been focused on BiVO_4_. It can be clearly seen in Figure 1 that research on BiVO_4_ photocatalysts has been active in recent years, indicating the important role that BiVO_4_ plays in the field of photocatalysis. Nevertheless, the photocatalytic behavior of pristine BiVO_4_ still remains unsatisfactory due to the serious recombination of photo-generated electron–hole pairs [16,17,18,19].

A summary of the recent advances in bismuth-based photocatalysts is of great importance to guide the development of this research topic in the near future. Some reviews have summed up the photocatalytic applications or water oxidation of bismuth-based photocatalysts [13,20]. However, the latest comprehensive and systematic review exclusively dedicated to BiVO_4_-based photocatalysts for photodegradation applications remains imperative, as it holds substantial significance for advancing the development of BiVO_4_-based photocatalysts in this field. To improve the photocatalytic degradation activity of BiVO_4_, various strategies including morphology control, crystal regulation, doping and heterojunction construction have been proposed to further improve the light absorption ability or the degree of charge carrier separation. In this review, we focused on BiVO_4_-based photocatalysts applied in the degradation of persistent organic pollutants in a water environment. We emphatically summarized the advancements in recent years regarding the modification strategies of BiVO_4_ and the relevant degradation mechanisms for efficient photocatalytic applications. We emphasized the influence of the regulation strategies on the relationship between structural features and photocatalytic activities of BiVO_4_. Finally, the challenges and perspectives of BiVO_4_-based photocatalysts for the degradation of persistent organic compounds were discussed and analyzed in depth.

## 2. Synthesis Method of BiVO_4_

The synthesis methods of BiVO_4_ significantly influence its photocatalytic performance. Different synthesis approaches modulate key characteristics such as crystal structure, morphology, specific surface area, defect concentration, and band structure. These factors influence the light absorption capacity and charge carrier separation efficiency of BiVO_4_. Hence, in order to synthesize BiVO_4_ with high catalytic activity, researchers have employed various synthesis methods including the hydrothermal method [21], co-precipitation method [22], sol–gel route [23], and microwave-assisted method [24] to prepare BiVO_4_.

Lin et al. [25] synthesized monoclinic BiVO_4_ photocatalysts using a hydrothermal method under different pH conditions. They discovered that BiVO_4_ prepared at a neutral pH (pH 7) demonstrated the highest photocatalytic performance towards Rhodamine B degradation. This enhanced photocatalytic activity is primarily due to the improved light absorption and efficient photogenerated charge separation, which are facilitated by the high exposure of {010} crystal facets, a closely packed structure, small particle size, and unique floating characteristics. Huang et al. [26] prepared different BiVO_4_ photocatalysts through a co-precipitation–hydrothermal synthesis method for the degradation of Rhodamine B and 2,4-Dichlorophenol. By adjusting the pH value and hydrothermal time, the ratio of monoclinic phase (m-BiVO_4_) to tetragonal phase (t-BiVO_4_) in the heterostructure can be successfully controlled. Notably, BiVO_4_ prepared at pH 7 and pH 0.5 with a hydrothermal time of 24 h exhibited the highest photocatalytic activity towards Rhodamine B and 2,4-Dichlorophenol degradation, respectively. The exposed crystal facets resulting from crystalline phase control are crucial reasons for the varying photocatalytic activities. BiVO_4_ with a monoclinic scheelite structure was synthesized successfully by Vidhya et al. using a sol–gel method combined with ultrasonication assistance [27]. And the photocatalyst showed great photocatalytic activity toward methylene blue due to the high surface area and better surface morphology. Shi et al. [28] developed a simple microwave-assisted method to prepare the branched BiVO_4_ nanocrystal photocatalyst, which exhibited great visible light response and high photocatalytic activity toward ciprofloxacin.

The aforementioned methods are capable of synthesizing BiVO_4_ photocatalysts with diverse structures and morphologies. The characteristics of BiVO_4_ directly determine its activity. Therefore, selecting an appropriate synthesis method is crucial when designing a BiVO_4_ photocatalyst in order to enhance its light absorption and charge separation efficiency.

## 3. Photocatalytic Degradation Mechanism of BiVO_4_

**Figure 2:**
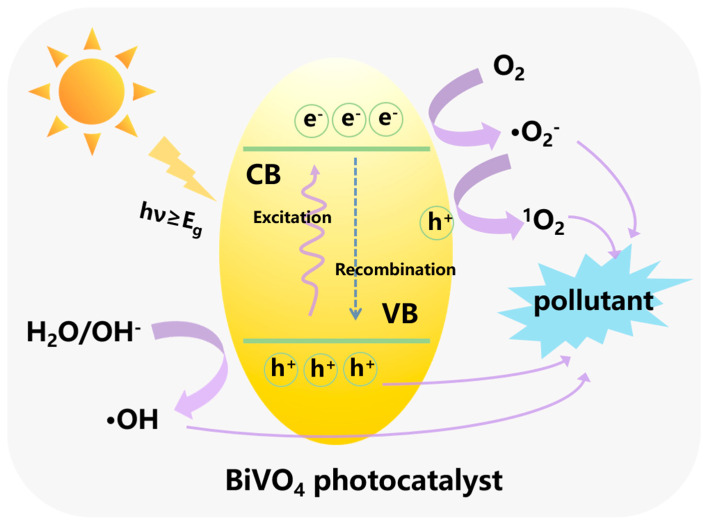
The photocatalytic degradation mechanisms of BiVO_4_.

Generally, there are three steps, including photon absorption, charge separation and surface chemical reactions, during the photocatalytic degradation process in organic compounds (Figure 2). Firstly, the photocatalyst absorbs photons with energy equivalent to or higher than its band gap, which causes the generation of photoinduced electrons in the valance band (VB) and holes in the conduction band (CB), respectively. Thereafter, the photogenerated carriers separate and migrate to the surface of the photocatalyst to participate in the next chemical reactions. The photogenerated electrons react with O_2_ to generate •O_2_^−^ radicals, whereas the photogenerated holes can oxidize H_2_O or OH^−^ to produce •OH radicals or react with •O_2_^−^ to produce ^1^O_2_. Finally, the pollutants can be decomposed into small molecules due to the highly oxidizing capability of •OH, •O_2_^−^and ^1^O_2_. The photogenerated holes can also directly participate in the reaction. In particular, the main active species in the reaction depend on the structures of different kinds of pollutants. The priority active species can be confirmed through various scavengers, such as benzoquinone, AgNO_3_, and so on. This process can be expressed by the following equations:

photocatalyst + hν → photocatalyst + h^+^ + e^−^

H_2_O/OH^−^ + h^+^ → •OH + H^+^

O_2_ + e^−^ → •O_2_^−^

•O_2_^−^+ h^+^ → ^1^O_2_

pollutants + •OH/•O_2_^−^/h^+^/^1^O_2_ → degradation products

## 4. General Strategy for Improving the Photocatalytic Degradation Activity of BiVO_4_

As the major challenges for effective photocatalytic degradation pollutants of BiVO_4_, light absorption and charge separation have attracted much attention during recent years. Accordingly, many strategies have been proposed to extend light absorption range or suppress the charge recombination of BiVO_4_. For example, morphology control has been applied to shorten the migration distance of photoinduced charge carriers [29,30]. Doping may change the electronic structure of BiVO_4_ and enhance its light absorption capacity [31,32]. Heterojunction construction has been fabricated to suppress the serious charge recombination of pure BiVO_4_ [33,34,35]. In addition, multiple strategies have been combined to improve the ability of light absorption and charge separation of the BiVO_4_ photocatalyst.

### 4.1. Morphology Control

It is well known that the photocatalytic activity of semiconductors not only depends on the chemical compositions but is also associated with the structure and morphology of photocatalysts [36,37]. In the photocatalytic process, the photogenerated holes and electrons produced by light absorption are excited in only a few femtoseconds, which is faster than the charge transfer and consumption of surface reaction. Therefore, it is preferable for charge recombination to occur before participating surface catalytic reactions. It is essential for photocatalysts to shorten the charge-migration distance to enhance charge separation. Morphology control has been considered as an effective method to overcome above bottlenecks. Different morphologies of BiVO_4_ have been reported in the last few decades, such as hollow spheres [38], nanosheet [39,40], starlike structure [41], needle-like structure [42], sandwich-like structure [43], and so on [44,45,46,47]. Table 1 lists the BiVO_4_-based photocatalysts with various morphologies applied in the photocatalytic degradation area in recent years.

**Figure 3:**
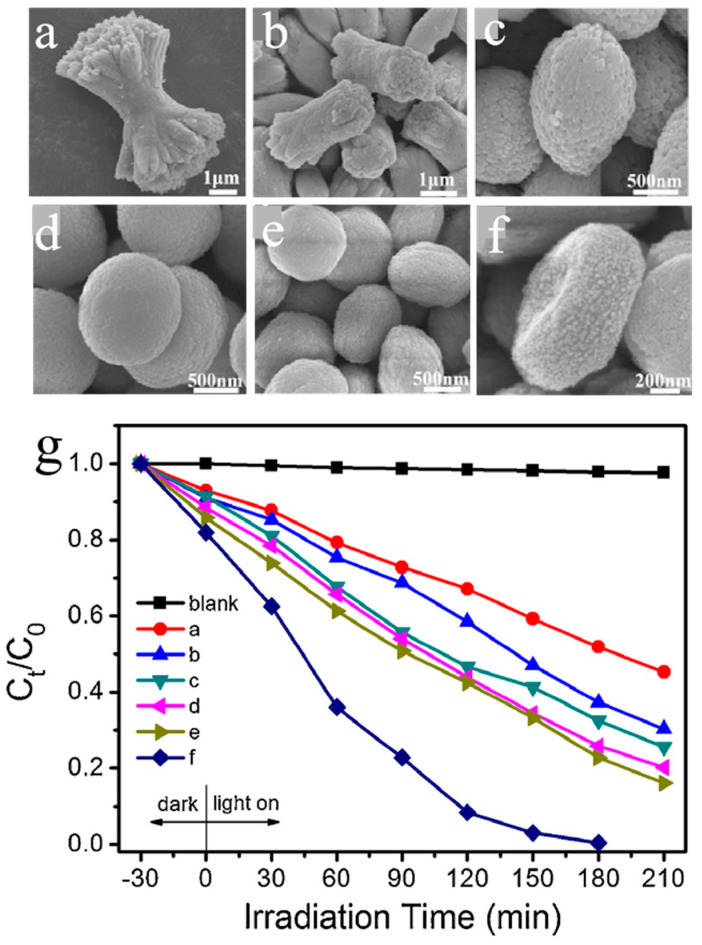
(**a**–**f**) SEM images of different morphological BiVO_4_ and (**g**) the corresponding photocatalytic performance towards Rhodamine B. Reproduced with permission from [48]. Applied Surface Science, 2018.

Wu et al. [48] successfully synthesized several morphological structures of BiVO_4_, including dumbbell, rod, ellipsoid, sphere, and cake-like, through a facile and surfactant-free hydrothermal method, as displayed in Figure 3. By just regulating the molar ratio of the starting precursor, the morphology of BiVO_4_ can be tuned easily. The photocatalytic activity of BiVO_4_ with different morphologies was evaluated by the degradation of Rhodamine B under visible light illumination, and the cake-like BiVO_4_ exhibited higher catalytic ability than other morphological BiVO_4_ owing to its special morphology, high surface area, and high charge separation efficiency. Similarly, Meng et al. [49] investigated the influence of KCl concentration on the morphology control and crystal growth of BiVO_4_. With the increase in KCl concentration, the morphology evolution of BiVO_4_ can be changed successively to rod-like, short rod-like form, cruciate structure, shuriken-like structure, and a tabular block. This is mainly because the concentration of Cl ions affected the crystal growth of BiVO_4_. Taking Rhodamine B as the target pollutant, the shuriken-like BiVO_4_ showed the highest photocatalytic performance, with a degradation efficiency of 94.7% after 240 min of irradiation. Hunge et al. [50] prepared a spherical-shaped BiVO_4_ and investigated the effect of deposition temperature on the morphology and optical properties. The spherical-shaped BiVO_4_ showed strong absorption in the visible region due to the special structure and exhibited good photocatalytic degradation ability for crystal violet dye.

It is obvious that untiring efforts have been taken to regulate the morphology of BiVO_4_ photocatalysts. However, the photocatalysts still yield unideal degradation efficiency. Hence, some works have combined morphology control and other strategies together to enhance the catalytic activities of photocatalysts. For example, Souza et al. [51] developed a microwave synthesis method to prepare BiVO_4_ nanoflowers decorated with Au nanoparticles applied in the photocatalytic degradation area. The nanoflower structure and the construction of the Au-BiVO_4_ heterojunction synergistically improved the charge separation of BiVO_4_, showing great potential for organic species degradation. Recently, hierarchically branched structures have drawn much attention due to their physicochemical characteristics. Abbood et al. [52] fabricated novel double-sided comb-like F/Ce co-doped BiVO_4_ micro/nanostructures applied in photocatalytic degradation and water oxidation. Based on morphology control and doping, the final photocatalyst exhibited excellent catalytic activity, in which doping enhanced the separation of photogenerated electrons and holes. Ma et al. [53] successfully synthesized ultrathin BiVO_4_ nanosheets of several nanometers in thickness in order to migrate the intrinsic issues of bulk BiVO_4_. BiVO_4_-Au-Cu_2_O nanosheets were subsequently constructed with satisfactory photocatalytic activity in the degradation of tetracycline with a removal rate of 80% within 30 min.

**Table 1:** Summary of BiVO_4_-based photocatalysts with various morphologies and corresponding photocatalytic activity.

BiVO_4_-Based Photocatalysts	Morphology	Light Source	Pollutant	Catalyst Dose (g/L)	Pollutant Concentration (mg/L)	Rate Constant (×10^−3^ min^−1^)	Degradation Efficiency	References
BiVO_4_	cake-like	350 W Xe lamp λ > 400 nm	RhB	3.33	/	/	99.5% (180 min)	[48]
BiVO_4_	shuriken-like	500 W Xe lamp λ > 420 nm	RhB	0.75	4.79	11.4	94.7% (240 min)	[49]
BiVO_4_	spherical	300 W Xe lamp λ > 420 nm	CV	1	18.65	3.528	98.21% (120 min)	[50]
Au-BiVO_4_	nanoflowers	UV-visible lamp	MB	/	0.5	/	94% (6 h)	[51]
F/Ce-BiVO_4_	double-sided comb-like	250 W mercury lamp λ > 420 nm	RhB	0.25	10	31.1	94.8% (100 min)	[52]
BiVO_4_	micro-leaves	simulated sunlight AM 1.5 G, 100 mW cm^−2^	MB	0.5	10	30.18	94% (90 min)	[54]
BiVO_4_	spindle-like	300 W Xe lamp λ > 420 nm	RhB	1	5	8.4	90.8% (180 min)	[55]
			TC	1	5	12.9	78.9% (180 min)	
BiVO_4_	nanoflowers	/	RhB	0.2	10	129.5	~100% (17 min)	[56]
BiVO_4_	nanocone	300 W Xe lamp λ > 420 nm	MB	/	10	/	99% (90 min)	[57]
BiVO_4_	shaving-like nanobelts	450 W tungsten lamp λ > 420 nm	MB	0.667	50	23.61	92.4% (120 min)	[58]
BiVO_4_	agglomerated particles	250 W Halogen lamp	MB	0.2	0.1	/	97% (90 min)	[59]
BiVO_4_	plate-like	300 W Xe lamp UV-visible light	CV	0.1	10	/	99.1% (90 min)	[60]
BiVO_4_	truncated-bipyramidal	15 W daylight lamp visible light	OTC	0.25	10	4.03	55% (240 min)	[61]
		Solar light		0.25	10	9.73	83% (240 min)	
BiVO_4_-Au-Cu_2_O	nanosheets	300 W Xe lamp λ > 420 nm	TC	1	30	163	80% (30 min)	[53]
Bi/BiVO_4_	chainlike hollow	500 W Xe lamp λ > 420 nm	RhB	1	10	/	91.8% (240 min)	[62]
BiVO_4_-CdS	hollow structure	300 W Xe lamp AM 1.5 G	TC	0.4	20	43.49	82.1% (25 min)	[63]
			CIP	0.4	10	14.56	84.1% (120 min)	

RhB, Rhodamine B; CV, crystal violet; MB, methylene blue; TC, tetracycline; OTC, oxytetracycline; CIP, ciprofloxacin.

### 4.2. Doping

Semiconductor doping is an effective approach to improve the intrinsic electronic properties of photocatalysts and reduce the recombination of photo-generated carriers, further improving the photocatalytic activity. Intermediate energy levels caused by doping will tune the optical properties and energy band structures of photocatalysts [64]. In addition, doping also affects the band structure by changing the Fermi level of semiconductors [65].

**Figure 4:**
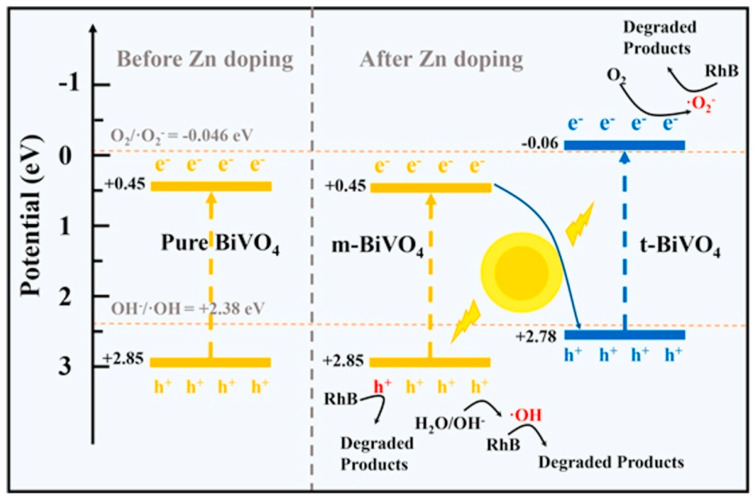
Possible mechanism of photocatalytic degradation of Rhodamine B by 2.5% Zn/BiVO_4_. Reproduced with permission from [66]. Optical Materials, 2021.

Recently, metal doping has been widely adopted to improve the photocatalytic activity of BiVO_4_. Zou et al. [66] successfully synthesized Zn-doped BiVO_4_ with a mixed crystal phase using a simple hydrothermal method. The replacement of Bi ions by Zn ions destroyed the original lattice of BiVO_4_, causing the crystal phase to change from monoclinic to tetragonal and the morphology to change from flakes to microspheres. The 2.5% Zn-doped BiVO_4_ showed the highest photodegradation rate of Rhodamine B, with 96% after 90 min, which was a great improvement compared with pure BiVO_4_. The reason was that the introduction of proper Zn ions induced the formation of a monoclinic-tetragonal heterostructure with staggered energy bands, which improved the mobility and separation of charge carriers. Thereafter, the photogenerated electrons and holes will participate in the radical reactions to further degrade the pollutants, as displayed in Figure 4. Peng et al. [31] doped Cu into BiVO_4_ to alter the electronic band structure to extend its light absorption ability. Meanwhile, the introduction of oxygen vacancies accompanied by doping can act as capture sites for charge carriers, thus suppressing charge recombination. Therefore, the Cu-doped BiVO_4_ distributed on montmorillonite exhibited improved photocatalytic performance, achieving a degradation rate of 99.32% for p-nitrophenol within 140 min.

Meanwhile, it should be noticeable that non-metal doping can also apply in the regulation of BiVO_4_ to improve its photocatalytic activity. For example, Regmi et al. [67] fabricated phosphate-doped BiVO_4_ via a microwave hydrothermal method. P-amino salicylic and ibuprofen were used to evaluate the photocatalytic activity of the as-prepared P-BiVO_4_ material under visible light illumination. Compared with pure BiVO_4_, the degradation efficiency of the doped BiVO_4_ improved 40%, and could reach 81% and 80% for p-amino salicylic and ibuprofen, respectively. DFT calculations indicated that the doping of P increased the electron density of states near the top of the valence band as well as improving the charge carrier mobility, which enhanced the charge separation of BiVO_4_. In addition, different charge densities and structural changes caused by bond length are also beneficial for performance improvements. Qin et al. [68] elaborated the effect of Br doping on the photocatalytic activity of BiVO_4_. The surface photovoltage spectroscopy results revealed that an impurity energy level came into being between the conduction band and the valance band of BiVO_4_ after the introduction of the Br atom, which promoted charge separation and further improved the photocatalytic activity. As a result, 3% Br-BiVO_4_ exhibited excellent photocatalytic performance for Rhodamine B and tetracycline and the abatement rate constant was 7 times higher than that of pure BiVO_4_. Trapping experiments demonstrated that superoxide free radicals and holes are the main active free radicals, while the proper amount of Br doping can form more active free radicals which benefited from the existence of an impurity energy level. Yang et al. [69] combined doping non-metal F element with the fabrication of a heterojunction to construct a novel F-BiVO_4_/g-C_3_N_4_/CdS dual S-scheme photocatalyst. The doping of F resulted in more active sites being exposed to the crystals and improved the light-harvesting capability of BiVO_4_, which contributed to the enhancement of the photocatalytic performance of the composites.

In addition to single-component doping, co-doping with two or more kinds of dopants has also been carried out to research the synergetic effects of different ions on photocatalysis. A Gd and Y co-doping BiVO_4_ photocatalyst with good crystallinity was reported by Noor et al. recently [70]. The co-doping regulated the structure of BiVO_4_ from monoclinic to tetragonal as well as the morphology from spherical to a nanorod-like shape. In addition, due to the rich energy levels and special electronic transition properties of Gd and Y ions, the optical properties and the degree of charge carrier separation were both changed. Compared with pure BiVO_4_ and mono-doped BiVO_4_, Gd/Y co-doping BiVO_4_ showed improved photocatalytic activity for methylene blue with 94% degradation efficiency within 90 min, which can be attributed to the synergistic effects of enhanced charge separation, high specific surface area, and good absorption ability. Similarly, Wang et al. [71] constructed Ag, B, and Eu tri-modified BiVO_4_ photocatalysts successfully using an ethylene glycol solgel and photo-deposition process. The optimized hybrid photocatalysts exhibited higher photocatalytic performance than that of single or co-modified samples toward methyl orange and tetracycline degradation. The photocatalytic mechanism was explored through free radical trapping experiments and photoluminescence tests. The lowest photoluminescence intensity of tri-modified BiVO_4_ indicated the enhanced charge separation of electron–hole pairs. In addition, the synergistic effects of Ag, Eu, and B caused BiVO_4_ to have a smaller band gap and increased visible light absorption. All these factors are beneficial for enhancing the photocatalytic performance of BiVO_4_. Table 2 summarizes BiVO_4_-based photocatalysts from recent years with different dopants, including single, co-doping, and doping combined with other strategies.

Although great progress has been achieved in improving the photocatalytic activity of BiVO_4_ through doping, the deeper mechanisms are still ambiguous due to the different roles the dopants played in semiconductors. The dopants may insert to interstice or replace the atoms of BiVO_4_, and the influence of these actions is quite different. Therefore, care should be taken to characteristic the dopants in detail to further reveal the working mechanisms.

### 4.3. Defects Engineering

Defects engineering has been widely accepted as a versatile approach to influence the charge carrier separation, light absorption ability and interfacial reactions of photocatalysts [94,95]. Introducing defects into BiVO_4_ also plays a critical role in improving its photocatalytic performance [96,97,98]. Jiang et al. [99] employed hydrogenation treatment to introduce oxygen vacancies on the surface of BiVO_4_ for the first time. The treatment led to the formation of a disordered amorphous layer, resulting from the creation of surface oxygen vacancies. X-ray photoelectron spectroscopy (XPS) and electron paramagnetic resonance spectra (EPR) were taken to observe the changes in oxygen vacancy concentration. At the same time, the narrowed band gap was also accompanied by enhanced light absorption ability. As a result, the hydrogenated BiVO_4_ exhibited excellent photocatalytic degradation efficiency toward tetracycline hydrochloride (98% for 30 min), much higher than that of pure BiVO_4_ (52% for 30 min). A series of test results showed that the existence of surface oxygen vacancies suppressed the recombination and facilitated the separation and migration of photogenerated charge carries. In addition, the carrier donor densities were also improved. Recently, an oxygen vacancy-rich BiVO_4_ hollow microsphere was successfully prepared with the assistance of sodium dodecyl benzene sulfonate by Li et al. [100]. The existence of oxygen vacancies largely elevated the separation efficiency of photogenerated charge carriers. Combined with morphology modulation, BiVO_4_ hollow microspheres with abundant oxygen vacancies exhibited excellent photocatalytic performance toward Rhodamine B.

**Figure 5:**
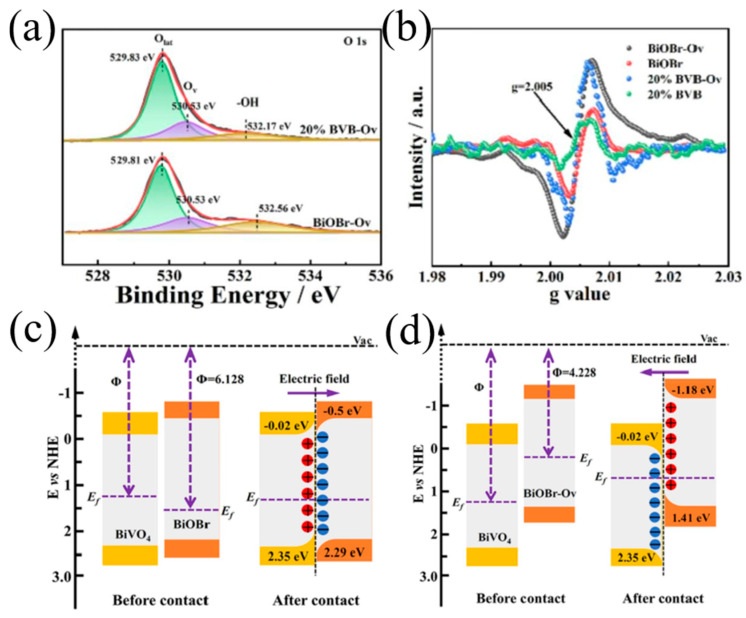
(**a**) XPS high-resolution spectra of O 1s, (**b**) EPR spectra for BiOBr-Ov, BiVO_4_, and 20% BVB-Ov composite. Schematic illustration of 20% BVB (**c**) and 20% BVB-Ov (**d**) heterostructure before and after contact. Reproduced with permission from [101]. Chemical Engineering Journal, 2022.

Liu et al. [101] introduced oxygen vacancy into BiVO_4_/BiOBr photocatalysts to investigate its influence on the interfacial charge transfer. XPS and EPR results proved the successful introduction of oxygen defects into the system (Figure 5a,b). Compared with pure 20% BiVO_4_/BiOBr, 20% BiVO_4_/BiOBr-O_V_ exhibited a higher photocatalytic degradation rate as well as excellent photostability toward oxytetracycline. In this system, the existence of oxygen vacancies modulated the relative Fermi level positions between BiVO_4_ and BiOBr, resulting in the transition from II-type to Z-scheme heterojunction (as shown in Figure 5c,d). Thus, more •O_2_^−^ is generated, promoting its participation in the degradation reactions. In addition, the introduction of oxygen vacancies inhibited the recombination of photogenerated charge carriers and enhanced charge separation, which was confirmed by relevant photoelectrochemical and photoluminescence results. Similarly, Li et al. [102] introduced oxygen vacancies in the system while constructing NiO/BiVO_4_. Oxygen vacancies enhanced the activation of molecular oxygen and acted as reactive sites for capturing photoinduced electrons, thereby facilitating the generation of •O_2_^−^. The synthesized catalysts showed great photocatalytic activities with the help of heterojunction structure and oxygen vacancies.

Though defects engineering is an attractive and effective strategy to reform the characteristic of BiVO_4_, it is still necessary to control the defect concentration precisely because it is a double-edged sword for photocatalysts. Balancing the advantages and disadvantages is a practical challenge for their further design and application.

### 4.4. Heterojunction Construction

The rational construction of heterostructures of two or more semiconductor materials can integrate the advantages of multiple components while improving photoinduced charge separation, expanding the visible light absorption range, and maintaining the high redox capacity of photocatalysts [103]. Therefore, semiconductor heterojunctions have become a research hotspot recently and have been widely applied in BiVO_4_-based photocatalysts. Due to their exceptional photocatalytic performance and unique phase structural characteristics, two-dimensional materials have emerged as a highly promising class of materials [104]. Therefore, we summarized BiVO_4_ heterostructure photocatalysts composited with two-dimensional materials and corresponding photocatalytic activity in Table 3. And Table 4 lists other partial heterojunction structures related to BiVO_4_ in recent years.

#### 4.4.1. Traditional Heterojunction

**Figure 6:**
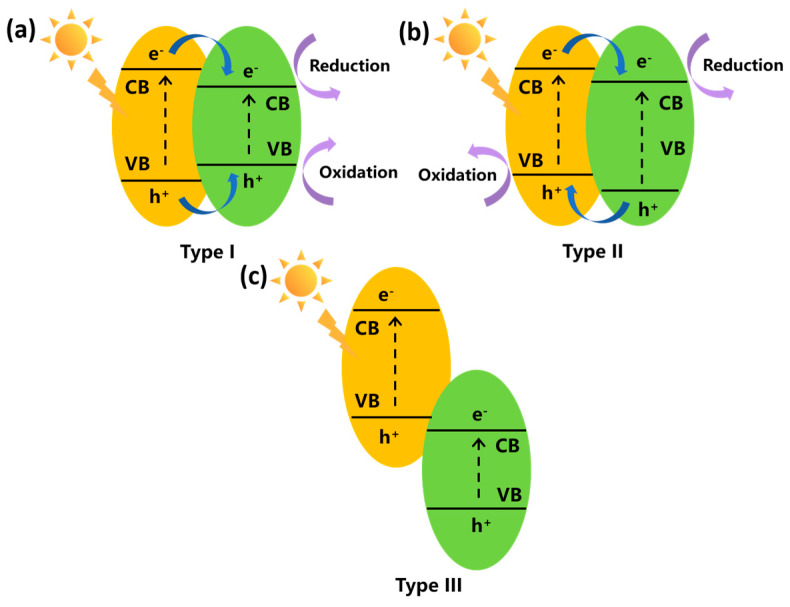
Schematic illustration of three kinds of traditional heterojunction photocatalysts: (**a**) type I, (**b**) type II, (**c**) type III.

There are three kinds of traditional heterojunction, including type I, type II, and type III, as displayed in Figure 6. For the type I heterojunction, the photogenerated electrons and holes both transfer from the semiconductor with higher conduction and lower valance band to another semiconductor. The electron–hole pairs will accumulate on one semiconductor and cannot separate effectively. Meanwhile, the redox ability of the composite photocatalyst will reduce because the photocatalytic reactions take place on the semiconductor with the lower redox potential [103]. The type II heterojunction is composed of two semiconductors with staggered energy bands. The photogenerated holes transfer to the semiconductor with the higher valance band and electrons to the semiconductor with the lower conduction band, resulting in a separation of charge carriers. Though the type III heterojunction is similar to the type II structure, the band level is so extreme that the bandgaps do not overlap [141]. There is no charge migration or separation between two semiconductors. Therefore, it is obvious that the type II heterojunction is the most suitable for improving the photocatalytic activity among these three kinds of traditional heterojunction.

**Figure 7:**
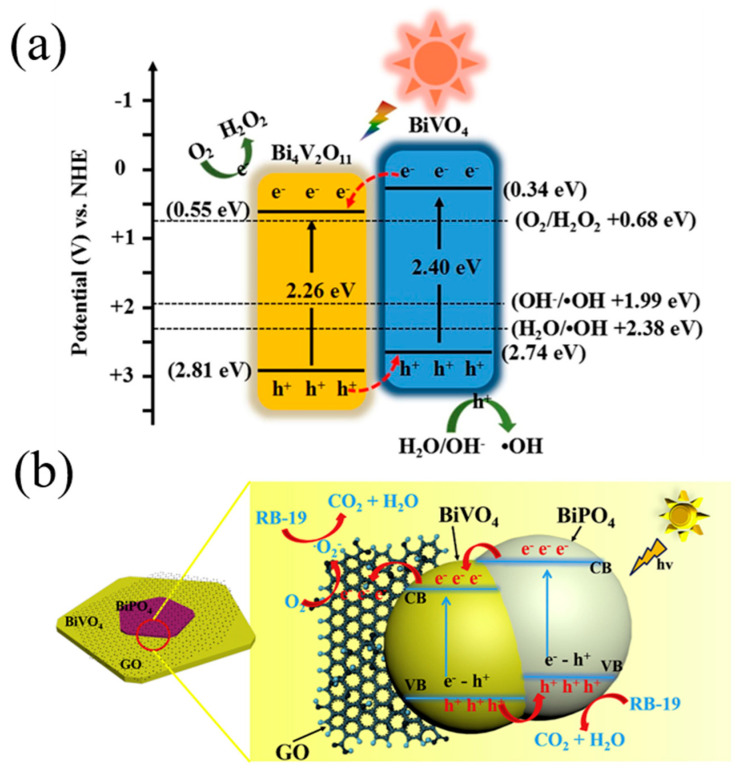
(**a**) Photocatalytic mechanism scheme of Bi_4_V_2_O_11_/BiVO_4_ heterostructure. Reproduced with permission from [123]. Applied Surface Science, 2021. (**b**) The possible photocatalytic reaction mechanism of BiVO_4_/BiPO_4_/GO for degradation under visible-light irradiation. Reproduced with permission from [110]. Journal of Cleaner Production, 2020.

Lin et al. [123] prepared a type II Bi_4_V_2_O_11_/BiVO_4_ heterostructure using by an easy one-pot hydrothermal method and investigated its photocatalytic degradation ability for Rhodamine B in detail. As displayed in Figure 7a, electron–hole pairs generated firstly from the photoexcited semiconductor components under light illumination. The holes transferred spontaneously from the valance band of Bi_4_V_2_O_11_ to that of BiVO_4_, and were then trapped by H_2_O or OH^−^ to produce •OH. Meanwhile, the electrons migrated from BiVO_4_ to the conduction band of Bi_4_V_2_O_11_ to react with O_2_ to generate H_2_O_2_. The efficient spatial transfer mode accelerated the separation of charge carriers, leading to an excellent photocatalytic activity of the Bi_4_V_2_O_11_/BiVO_4_ type II heterostructure. As a result, the photodegradation rate of Bi_4_V_2_O_11_/BiVO_4_ towards Rhodamine B was 10.86 times higher than that of pure BiVO_4_. Li et al. [142] synthesized a continuous type II Ag_3_VO_4_-BiVO_4_/C_3_N_4_ heterojunction for the effective removal of Cr (VI). The formation of an internal electric field between Ag_3_VO_4_, BiVO_4_, and C_3_N_4_ improved the charge separation efficiency. Isolated oxidation and reduction reactions can occur due to suitable band alignments, which also help to suppress charge recombination. Yang et al. [109] successfully developed a novel 3D/2D/2D BiVO_4_/FeVO_4_@rGO heterojunction structure using the one-step hydrothermal method, which significantly improved the degradability towards tetracycline and the reduction ability of Cr (VI). As a typical two-dimensional material, rGO exhibits a high specific surface area, exceptional electrical conductivity, and abundant active reaction sites. Incorporating rGO into photocatalysts can significantly enhance the electron migration efficiency. Due to the successful construction of the heterojunction, the nanochannels formed in the interfacial contact, which can accelerate charge separation and enhance light absorption. Therefore, BiVO_4_/FeVO_4_@rGO exhibited higher photocatalytic performance than that of pure FeVO_4_, BiVO_4_, and binary BiVO_4_/FeVO_4_. Similarly, a BiVO_4_/BiPO_4_/GO photocatalyst was synthesized successfully by Wang and co-authors [110]. The formation of a type II heterojunction between BiVO_4_ and BiPO_4_ enhanced electron–hole pair separation because photogenerated holes and electrons migrated in an opposite direction, as shown in Figure 7b. Meanwhile, GO can collect the photogenerated electrons due to its excellent conductivity, thus further enhancing charge separation. Based on the above factors, the BiVO_4_/BiPO_4_/GO photocatalyst presented outstanding photocatalytic activity with 99% degradation efficiency toward RB-19 dye within 60 min.

#### 4.4.2. p-n Heterojunction

Although the type II heterojunction can improve charge separation in space, the photocatalytic degradation efficiency is still limited due to the fast electron–hole recombination. Thus, p-n heterojunction structures were proposed, which can promote electron–hole migration across the heterojunction by providing an additional electric field [143].

An effective p-n heterojunction consists of p-type and n-type semiconductors. The internal electric field comes into being on the interface between these two kinds of semiconductors, which accelerates electrons’ transfer to the conduction band of n-type semiconductors and holes to the valence band of p-type semiconductors. Compared with type II heterojunction, the charge separation efficiency improved due to the synergistic effect of band alignment and the internal electric field [103]. Therefore, the p-n heterojunction exhibited improved photocatalytic performance and has been widely applied in the construction of BiVO_4_-based composite photocatalysts [114,115,119,132,144,145,146].

For example, Li et al. [119] constructed BiOCl/BiVO_4_ p-n heterojunction photocatalysts containing vacancies applied in the photocatalytic degradation of phenol. The synergistic effect of oxygen vacancies and the built-in field resulted in a two-fold increase in photocatalytic activity towards phenol compared to pure BiVO_4_. Wei et al. [114] fabricated novel p-n heterojunction Ag_2_S/BiVO_4_ photocatalysts applied in the photocatalytic degradation of tetracycline. Compared with pure Ag_2_S and BiVO_4_, the binary composite photocatalyst exhibited improved photocatalytic activity with 90.2% degradation efficiency in 150 min, which can be attributed to the facilitated charge transfer caused by the heterostructure. In addition, the lifetime of Ag_2_S/BiVO_4_ also increased and thus weakened the photoinduced electron–hole pairs’ recombination. A micro-nano spherical p-n heterojunction CoO/BiVO_4_ was synthesized successfully by Wang et al. [115] for improving the photocatalytic activity toward tetracycline degradation. The modification of CoO avoided deactivation and formed a p-n heterostructure with BiVO_4_. Therefore, the photogenerated electron–hole pairs can migrate and separate effectively under the built-in electric field with an apparent quantum efficiency of 0.54%. The degradation mechanism investigated using free radical trapping and electron spin resonance experiments proved that •O_2_^−^ and h^+^ are the main active species in this system.

#### 4.4.3. Z-Scheme Heterojunction

The transfer path of photogenerated charge carriers in the Z-Scheme heterojunction is very different from that in the type II heterojunction. In the type II heterostructure, photogenerated electrons usually transfer into the conduction band with weaker reduction ability, while holes migrate into the valence band with weaker oxidation ability, which weakens the redox ability of single components in the composite photocatalysts [147]. In order to overcome this shortcoming, Z-Scheme heterojunction photocatalysts were put forward by Bard. et al. in 1979, inspired by the photosynthesis of green plants [148]. Compared with the conventional heterojunction, Z-scheme heterojunction can not only separate the photoinduced charges effectively but also maintain the strong redox ability of the charges.

**Figure 8:**
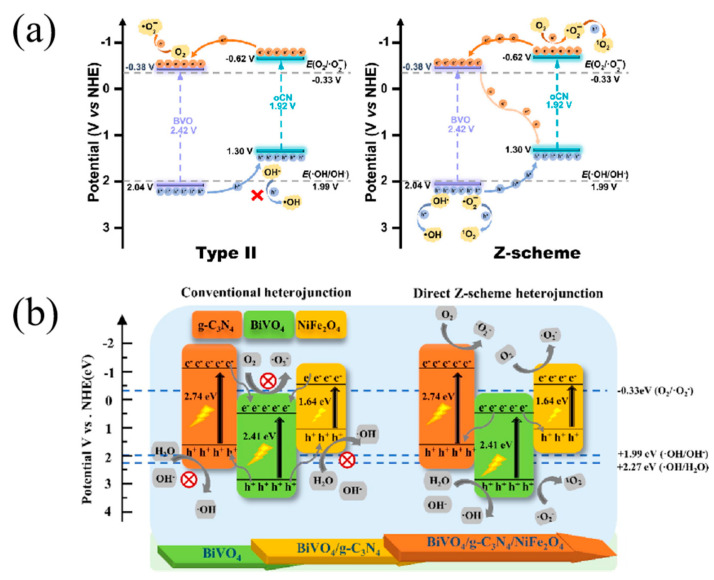
(**a**) The possible charge transfer pathways in BiVO_4_/O-g-C_3_N_4_ composites: type II and Z-scheme heterojunction. Reproduced with permission from [135]. Journal of Water Process Engineering, 2024 (**b**) The charge transfer path of the dual Z-scheme heterojunction BiVO_4_/g-C_3_N_4_/NiFe_2_O_4_. Reproduced with permission from [138]. Chemical Engineering Journal, 2021.

Recently, hierarchical columnar ZnIn_2_S_4_/BiVO_4_ Z-scheme heterojunctions have been designed through an in situ method by Xia et al. [121]. The confined built-in electric field between ultrathin ZnIn_2_S_4_ nanosheets and BiVO_4_ pillars accelerated the migration of charge carriers, especially at heterointerfaces. Thus, the ZnIn_2_S_4_/BiVO_4_ exhibited excellent photocatalytic properties in the mineralization of antibiotics. Li et al. [135] successfully fabricated a Z-scheme BiVO_4_/O-g-C_3_N_4_ photocatalyst enriched with oxygen vacancies and interfacial chemical bonds. The formation of metal-O (V-O and Bi-O) bonds connected two monomers tightly and accelerated charge transfer. Meanwhile, the synergistic effect of oxygen vacancies and interfacial chemical bonds largely improved the charge separation efficiency. Thus, the nanocomposites exhibited great photocatalytic degradation activity toward tetracycline. Figure 8a showed the possible charge transfer pathways in BiVO_4_/O-g-C_3_N_4_ composites. Based on radical analysis, a Z-scheme heterojunction was confirmed. The photogenerated electrons in the conduction band of BiVO_4_ can react with O_2_ to generate •O_2_^−^, followed by further oxidation of holes to produce ^1^O_2_^−^, while the holes in the valance band combined with OH^−^ to generate •OH. The novel Z-scheme heterojunction can also be applied in different practical water samples with stable photocatalytic activity, indicating excellent practical application prospects.

Moreover, Zhao et al. [138] devised and constructed a magnetic dual Z-scheme system of a BiVO_4_/g-C_3_N_4_/NiFe_2_O_4_ heterojunction for the photocatalytic degradation of OFL under visible-light irradiation. In this dual Z-scheme heterojunction system, the photogenerated electrons migrated from BiVO_4_ to the valance band of g-C_3_N_4_ and NiFe_2_O_4_, followed by recombination with photoinduced holes (as displayed in Figure 8b). Thus, the electrons in the conduction band of g-C_3_N_4_ and NiFe_2_O_4_ and holes in the valance band of BiVO_4_ can separate more effectively compared with the conventional heterojunction. As a consequence, the BiVO_4_/g-C_3_N_4_/NiFe_2_O_4_ exhibited the highest degradation rate constant for OFL removal, accompanied by good reusability and stability. Relevant experiments indicated that the dual Z-scheme heterojunction enhanced the internal interaction between different semiconductors, resulting in enhanced charge separation, improved visible-light response, and high redox capacity.

#### 4.4.4. S-Scheme Heterojunction

**Figure 9:**
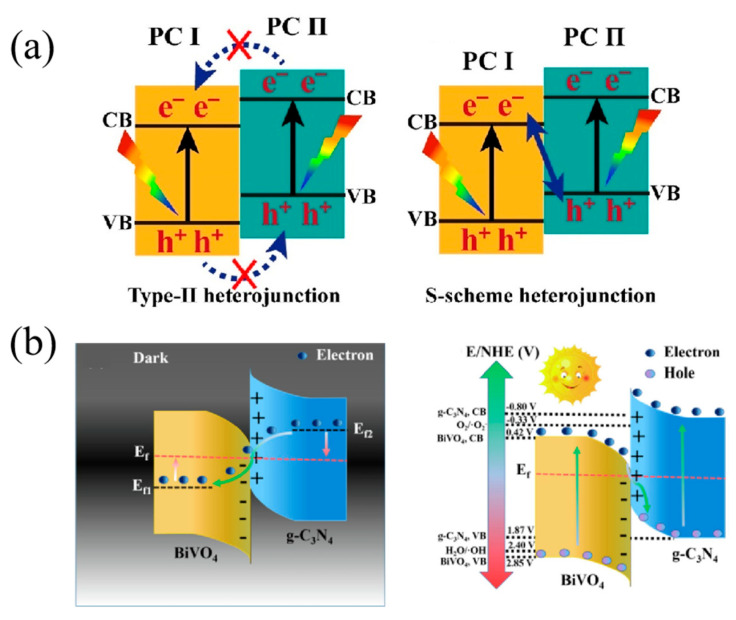
(**a**) The schematic diagrams of charge transfer in a conventional type-II heterojunction and S-scheme heterojunction. Reproduced with permission from [149]. Applied Catalysis B:environment and Energy, 2019. (**b**) Schematic illustration of the electron transport mechanism of BiVO_4_/g-C_3_N_4_ heterojunction photocatalyst under dark conditions and simulated sunlight irradiation. Reproduced with permission from [128]. Separation and Purification Technology, 2022.

Recently, a novel step-scheme (S-scheme) heterojunction was proposed by Yu’s group and is illustrated in Figure 9a [149,150,151]. Typically, the S-scheme heterojunction is composed of an oxidation photocatalyst (OP) and a reduction photocatalyst (RP) with staggered band structures. The valance band position of OP is more positive and the conduction band of RP is more negative, which ensures the strong redox ability of the composite photocatalysts. As displayed in Figure 9a, different from the type II heterojunction, the migration route of photogenerated electrons is more like a “step” in macroscopic or “N” in microscopic. With the help of the internal electric field, band bending and Coulombic attraction, which were key factors to enhance charge separation, the S-scheme heterojunction photocatalysts have exhibited great application potential in photocatalytic areas in recent years [32,69,96,98,102,136].

For example, Jin et al. [92] combined La-doped BiVO_4_ with g-C_3_N_4_ nanosheets by self-assembly to build a S-scheme heterojunction (La-BiVO_4_@CN). The construction of the S-scheme heterostructure facilitated photogenerated carrier separation and possessed good redox ability to activate dissolved oxygen. In addition, the interaction between La-BiVO_4_ and g-C_3_N_4_ enhanced light harvesting of the composites. Relevant experimental and theoretical results indicated that the doping of La in BiVO_4_ was beneficial to generate oxygen vacancy to facilitate O_2_ adsorption and activation. Therefore, the La-BiVO_4_@CN exhibited excellent photocatalytic degradation ability of tetracycline. Similarly, Chen et al. [128] successfully constructed the BiVO_4_/g-C_3_N_4_ S-scheme heterojunction via a green and rapid microwave method by depositing BiVO_4_ on the surface of g-C_3_N_4_. In this reaction system, acetic acid can take the place of nitric acid to induce the nucleation of Bi^3+^ and VO_4_^3−^. The ethylene glycol can shorten the reaction time and be recycled. When BiVO_4_ and g-C_3_N_4_ were combined, a new Fermi level was formed to reach equilibrium, thus forming the internal electric field and energy band bending at the interface (Figure 9b). Due to the construction of the S-scheme heterojunction, the utilization of photoinduced charge carriers largely improved, resulting in excellent photocatalytic degradation activity of the composites. The final photocatalyst BV/CN_60_-R_20_ showed great photocatalytic performance toward glyphosate, enhanced by 14.5 and 9.7 times compared to pure BiVO_4_ and g-C_3_N_4_, respectively. Recently, a novel peanut-like Zn_0.5_Cd_0.5_S/BiVO_4_ S-scheme heterostructure photocatalyst was successfully fabricated via a two-step hydrothermal method by Zhou and his cooperators [136]. The band bending at the interface caused the formation of a built-in electric field, which inhabits the charge recombination and prolongs the carrier lifetime. The optimized photocatalyst showed exceptional photocatalytic performance, with a tetracycline degradation efficiency of up to 99.52%.

It is obvious that many efforts have been made to construct efficient BiVO_4_-based heterojunctions in recent years. Of course, significant challenges still remain in preparing high-quality heterostructure BiVO_4_-based photocatalysts using much more simple and economic methods. In addition, systematic investigations of the transfer pathway of charge carriers are further needed because there is no direct evidence to display the actual migration pathway at the interface.

## 5. Conclusions and Perspectives

In conclusion, this review discussed the development over the past years of BiVO_4_-based photocatalyst-related photocatalytic degradation for organic pollutants. The advantages of a BiVO_4_ semiconductor, e.g., narrowed band gap and unique electronic structure, have been well acknowledged. However, the single semiconductor still suffered from several limitations for particular applications. In this review, we summarized a concise appraisal of the current achievements of BiVO_4_-based photocatalysts from the aspects of morphology control, doping, defect engineering, and heterojunction construction, demonstrating the important role of BiVO_4_ displayed in the photocatalytic area. Among these modification strategies, heterojunctions enhance photocatalytic performance by promoting charge separation and broadening the light absorption range, while defect engineering improved it by enhancing light absorption and surface reaction activity, both of which are highly effective in boosting photocatalytic performance. However, the specific effects need to be comprehensively analyzed based on experimental conditions and target applications, and the synergistic use of multiple strategies typically yields better results.

Photocatalytic degradation of pollutants has entered a dramatically changing era in terms of material construction, mechanism research and practical application. We believe that BiVO_4_-based photocatalysts are and will continue to be preferred choices for the photodegradation of pollutants. Despite the rapid progress in this field, some fundamental issues and challenges must be addressed to achieve excellent practical applications.

(1) Although there are currently some methods for synthesizing BiVO_4_-based materials, it is necessary to develop more efficient, simple and economic methods to prepare BiVO_4_-based photocatalysts on a large scale for practical applications. Further progress is imperative in exploring novel synthesis methods and regulation strategies to suppress charge recombination with the sustainable development of nanoscience and nanotechnology.
(2) Over the past few years, significant advancements have been made in the development and research of BiVO_4_-based heterojunctions. It can be concluded that, in comparison to traditional heterojunctions, Z-Scheme and S-Scheme heterojunctions are capable of simultaneously achieving high redox capacity and efficient separation of photogenerated charges. Their distinctive charge transfer mechanisms endow the photocatalysts with enhanced reactivity and stability in photocatalytic reactions. Significant efforts should be devoted to designing BiVO_4_-based binary and multivariate heterojunctions with enhanced photocatalytic activity.
(3) The migration pathway of photogenerated electrons and holes needs to be further investigated to reveal the photocatalytic reaction mechanisms in depth, especially at the atomic level, which is beneficial for improving the degradation efficiency.
(4) The photo-stability of BiVO_4_-based photocatalysts is an important factor for practical applications. The question of how to inhibit photo-corrosion to a great extent and prolong the lifetime of photocatalysts should be given great consideration.
(5) For practical applications, the matrix effects may cause a negative influence on pollutant removal during the photocatalytic process. The complexity of the actual samples requires that BiVO_4_-based photocatalysts with excellent anti-influence ability should be further developed.

## Figures and Tables

**Table 2:** Summary of BiVO_4_-based photocatalysts with different dopants and corresponding photocatalytic activity.

BiVO_4_-Based Photocatalysts	Light Source	Pollutant	Catalyst Dose (g/L)	Pollutant Concentration (mg/L)	Rate Constant (×10^−3^ min^−1^)	Degradation Efficiency	References
Zn-BiVO_4_	300 W Xe lamp	RhB	1	5	/	96% (90 min)	[66]
Cu-BiVO_4_	500 W Xe lamp	PNP	0.4	20	30.1	99.32% (140 min)	[31]
P-BiVO_4_	A 150 W short arc lamp λ > 420 nm	PAS	1	20	5	81% (300 min)	[67]
		IBP	1	20	8	80% (180 min)	
Br-BiVO_4_	500 W Xe lamp λ > 420 nm	RhB	1	10	5.6	76.9% (240 min)	[68]
		TC	1	20	42	78.9% (30 min)	
La-BiVO_4_	visible light	RhB	0.6	10	0.18	94.9% (180 min)	[72]
Nd^3+^-BiVO_4_	500 W Xe lamp	MB	1	10	/	89.22% (90 min)	[73]
Co-BiVO_4_	LED lamp	RhB	0.5	15	1.882	96.78% (180 min)	[74]
Fe-BiVO_4_	500 W Xe light maximum 470 nm	MB	/	5	4.031	~100% (180 min)	[75]
Al^3+^-BiVO_4_	500 W Xe lamp λ > 300 nm	RhB	0.5	10	12.03	96% (90 min)	[76]
Ti-BiVO_4_	32 W visible light bulb	TC	1	15	/	78.5% (240 min)	[77]
Eu-BiVO_4_	300 W Xe lamp λ > 420 nm	TC	0.5	20	/	91.4% (150 min)	[78]
Cu-BiVO_4_	150 W short arc lamp λ > 420 nm	MB	1.43	20	8	95% (150 min)	[79]
		IBP	1.43	20	/	75% (90 min)	
Gd^3+^-BiVO_4_	LED lamp	BPA	1	10	7.23	77.02% (180 min)	[80]
		BPS	1	10	/	44.36% (180 min)	
		BPAF	1	10	/	74.11% (180 min)	
N-BiVO_4_	A 150 W short arc lamp λ > 420 nm	IBP	1	20	13.1	90% (150 min)	[81]
F-BiVO_4_	400 W halogen lamp λ > 420 nm	Gly	0.75	16.909	/	~100% (6 h)	[82]
In/Fe-BiVO_4_	300 W Xe lamp λ > 420 nm	TC	0.2	10	15.99	82% (120 min)	[83]
F/Ce-BiVO_4_	250 W mercury lamp λ > 420 nm	RhB	0.25	10	3.11	94.8% (100 min)	[52]
Gd/Y-BiVO_4_	500 W Xe light 100 mW/cm^2^	MB	1	10	19.02	94% (90 min)	[70]
S/W-BiVO_4_	150 W short arc lamp λ > 420 nm	NPX	1	10	6.7	76.5% (180 min)	[84]
Ag/Yb-BiVO_4_	Solar light	CV	1	7	/	94.57% (120 min)	[85]
Ag/Cu-BiVO_4_	500 W Xe lamp λ > 400 nm	MB	0.1	5	11.9	87% (150 min)	[86]
Ag/B/Eu-BiVO_4_	250 W metal halide lamp λ > 420 nm	MO	0.2	15	39.5	~90% (50 min)	[71]
		TC	0.2	20	29	~75% (50 min)	
W-BiVO_4-x_/rGO	300 W Xe lamp λ > 420 nm	CIP	1	10	43.77	93.6% (60 min)	[87]
		CTC	1	10	32.8	86.8% (60 min)	
F-BiVO_4_/g-C_3_N_4_/CdS	500 W Xe lamp	CIP	1	20	64.55	90% (30 min)	[69]
W- BiVO_4_/g-C_3_N_4_	220 W tungsten filament light bulb	PM	0.43	2	18	91% (120 min)	[88]
		ASA	0.43	2	16	86% (120 min)	
Co-BiVO_4_/Bi_2_O_3_	300 W Xe lamp λ > 420 nm	CTC	0.3	30	72.67	90.5% (30 min)	[89]
		MBT	0.2	20	85.7	97.7% (40 min)	
g-C_3_N_4_-Eu-BiVO_4_	250 W halogen lamp λ > 410 nm	RhB	0.2	10	77.9	98% (50 min)	[90]
Cu-BiVO_4_-g-C_3_N_4_	350 W Xe light	BPA	0.4	20	42.6	~100% (90 min)	[91]
La-BiVO_4_-g-C_3_N_4_	500 W Xe arc lamp λ > 420 nm	TC	0.25	20	82.1	81.7% (75 min)	[92]
g-C_3_N_4_/Na-BiVO_4_/PMS	300 W Xe lamp λ > 420 nm	TC	0.2	20	109.99	98.2% (40 min)	[93]

RhB, Rhodamine B; CV, crystal violet; MB, methylene blue; TC, tetracycline; CIP, ciprofloxacin; MO, methyl orange; PNP, p-nitrophenol; IBP, ibuprofen; PAS, p-amino salicylic acid; BPA, bisphenol A; BPS, bisphenol S; BPAF, bisphenol AF; Gly, glyphosate; NPX, naproxen; CTC, chlortetracycline; PM, pendimethalin; ASA, Aspirin; MBT, mercaptobenzothiazole.

**Table 3:** Summary of BiVO_4_ heterostructure photocatalysts composited with two-dimensional materials and corresponding photocatalytic activity.

BiVO_4_-Based Heterostructure Photocatalysts	Light Source	Pollutant	Catalyst Dose (g/L)	Pollutant Concentration (mg/L)	Rate Constant (×10^−3^ min^−1^)	Degradation Efficiency	References
BiVO_4_/g-C_3_N_4_	300 W Xenon arc lamp λ > 420 nm	RhB	0.5	20	41.0	~100% (60 min)	[105]
BiVO_4_-Bi_2_WO_6_/g-C_3_N_4_	300 W Xe lamp λ > 420 nm	RhB	1	20	58	96.7% (60 min)	[106]
		TC	1	20	48	94.8% (60 min)	
BiVO_4_/h-BN	400 W Xe lamp visible light	RhB	0.8	15	12.1	93.3% (60 min)	[107]
			0.8	15	29.2	92.9% (60 min)	
			0.8	15	18.2	94.1% (60 min)	
			0.4	15	37.7	90.1% (60 min)	
BiVO_4_/SnS_2_	natural sunlight	OFL	0.2	10	16.4	93.7% (150 min)	[108]
		TC	0.2	10	10	80.8% (150 min)	
BiOI/BiVO_4_	Sunlamp λ > 400 nm	RhB	0.6	5	46.26	97% (75 min)	[29]
BiVO_4_/FeVO_4_@rGO	1 KW Xe lamp λ > 420 nm	TC	0.6	30	31.37	91.5% (90 min)	[109]
BiVO_4_/BiPO_4_/GO	Xe lamp	RB-19 dye	0.6	80	35.02	99% (60 min)	[110]
AgVO_3_/rGO/BiVO_4_	250 W Xe lamp	NFC	/	/	18.7	96.1% (90 min)	[111]
BiOCl (110)/NrGO/ BiVO_4_	150 W Xe lamp λ > 420 nm	SMZ	0.05	15	/	96.9% (60 min)	[112]

RhB, Rhodamine B; TC, tetracycline; OFL, ofloxacin; NFC, norfloxacin; SMZ, sulfamethazine.

**Table 4:** Summary of BiVO_4_-based heterostructure photocatalysts and corresponding photocatalytic activity.

BiVO_4_-Based Heterostructure Photocatalysts	Light Source	Pollutant	Catalyst Dose (g/L)	Pollutant Concentration (mg/L)	Rate Constant (×10^−3^ min^−1^)	Degradation Efficiency	References
BiVO_4_/BiOBr	300 W Xe lamp λ > 420 nm	OTC	/	10	43.45	91% (60 min)	[101]
BiVO_4_/0.6CdS	500 W Xe lamp λ > 420 nm	TC	0.5	20	29	90.6% (80 min)	[113]
Ag_2_S/BiVO_4_	500 W Xe lamp λ > 420 nm	TC	0.4	20	10	90.2% (150 min)	[114]
CoO/BiVO_4_	300 W Xe lamp λ > 420 nm	TC	0.6	40	22.94	87.3% (90 min)	[115]
CuS/BiVO_4_	300 W Xe lamp λ > 420 nm	CIP	1	10	21.51	86.7% (90 min)	[116]
ZnO/BiVO_4_	15 W cool daylight twist-shape bulb	SA	1	10	4.9	59% (180 min)	[117]
		RB5	1	10	13.2	92% (180 min)	
AgIO_3_/BiVO_4_	300 W Xe lamp λ > 420 nm	CBZ	1	5	120.2	97.86% (60 min)	[118]
BiOCl/BiVO_4_	300 W Xe lamp λ > 420 nm	Ph	0.4	10	4.56	64% (240 min)	[119]
B-TiO_2_/BiVO_4_	300 W Xe lamp λ > 420 nm	OTC	0.5	20	47.2	89.3% (120 min)	[120]
ZnIn_2_S_4_/BiVO_4_	250 W Xe lamp	TC	0.5	20	6.82	60% (180 min)	[121]
BiVO_4_/TiO_2_	300 W Xe lamp	RhB	1	/	21	~95% (120 min)	[122]
Bi_4_V_2_O_11_/BiVO_4_	400 W metal halide lamp	RhB	0.1	15	25	91.1% (90 min)	[123]
g-C_3_N_4_/BiVO_4_	250 W Xe lamp λ > 420 nm	MO	0.2	20	96.7	94.2% (50 min)	[124]
ZnIn_2_S_4_/BiVO_4_	300 W Xenon lamp λ > 420 nm	TC	0.2	20	20.44	87.71% (75 min)	[125]
BiVO_4_/Ag_3_VO_4_	50 W 410 nm LED	MB	1	50	55.88	93.48% (50 min)	[126]
Bi-MOF/BiVO_4_	300 W Xe lamp λ > 420 nm	TC	0.2	10	129.5	96.2% (30 min)	[127]
BiVO_4_/g-C_3_N_4_	300 W Xe lamp AM 1.5 G	Gly	0.5	10	15	87% (120 min)	[128]
BiVO_4_/BaSnO_3_@HNTs	200 W LED lamp	MB	1	20	19.26	94.1% (120 min)	[129]
Ag/Ag_2_CO_3_/BiVO_4_	500 W Xe lamp	TC	0.4	20	18.6	94.9% (150 min)	[130]
Pd@Bi_2_Ru_2_O_7_/BiVO_4_	300 W Xe lamp	RhB	1	10	124	100% (30 min)	[131]
BiVO_4_/PANI	500 W tungsten halogen lamps	p-NP	0.05	20	7.2	98.25% (250 min)	[132]
Ag/p-Ag_2_S/n-BiVO_4_	500 W Xe lamp λ > 420 nm	OTC	0.4	15	41.1	99.8% (150 min)	[133]
Ag/AgNbO_3_/BiVO_4_	65 W energy-saving lamp	LEF	0.6	20	23.5	74.46% (120 min)	[134]
BiVO_4_/O-g-C_3_N_4_	300 W Xe lamp λ > 420 nm	TC	30	20	126.6	99.8% (60 min)	[135]
Zn_0.5_Cd_0.5_S/BiVO_4_	300 W Xe lamp	TC	10	/	/	99.52% (120 min)	[136]
δ-Bi_2_O_3_/m-BiVO_4_/g-C_3_N_4_	500 W halogen lamp	TC	1	20	/	92% (60 min)	[137]
BiVO_4_/g-C_3_N_4_/NiFe_2_O_4_	300 W Xe lamp λ > 420 nm	OFL	1	10	135.3	93.8% (20 min)	[138]
InVO_4_/CuBi_2_O_4_/BiVO_4_	300 W Xe lamp	TC	1	20	7.9	87.1% (180 min)	[139]
g-C_3_N_4_/BiVO_4_ (040)/In_2_S_3_	300 W Xe lamp	TC	0.8	40	6.1	68.2% (180 min)	[140]

RhB, Rhodamine B; MB, methylene blue; TC, tetracycline; OTC, oxytetracycline; CIP, ciprofloxacin; MO, methyl orange; SA, salicylic acid; RB5, reactive black 5; Gly, glyphosate; CBZ, carbamazepine; Ph, phenol; p-NP, p-nitrophenol; LEF, levofloxacin; OFL, ofloxacin.

## Data Availability

No new data were created or analyzed in this study.
